# POMsDB: a QM derived molecular properties and parameters database for Polyoxometalates

**DOI:** 10.1038/s41597-026-07770-7

**Published:** 2026-08-24

**Authors:** Mireia Segado-Centellas, Albert Masip-Sánchez, Carles Bo

**Affiliations:** 1 Departament de Qumica Fsica i Inorgànica, Universitat Rovira i Virgili (URV), c/ Marcel·l´ı Domingo s/n, 43007 Tarragona, Spain; 2 Departament de Física, Escola Tècnica Superior d’Enginyeria Industrial de Barcelona (ETSEIB), Universitat Politècnica de Catalunya. BarcelonaTech (UPC), Campus Sud, Edif. PG, Av. Diagonal, 647, 08028 Barcelona, Spain; 3 Institute of Chemical Research of Catalonia (ICIQ), Barcelona Institute of Science & Technology (BIST), Avda. Pasos Catalans, 16, 43007 Tarragona, Spain

## Abstract

Polyoxometalates (POMs) are an important class of anionic inorganic compounds because of their rich structural chemistry and properties. POMsDB is a freely accessible database for POMs that collects molecular properties (optimized atomic coordinates, energies, IR spectrum, atomic charges) and force-field (FF) parameters to run molecular dynamic (MD) simulations in common MD programs. FF parameters in this database consist of nonbonded (atomic point charges, Lennard-Jones (LJ)) and bonded parameters. Three distinct types of DFT derived atomic charges and two sets of LJ parameters could be selected. In addition, two definitions of bonding intramolecular parameters are available: one where the POM behaves as a rigid object, and the other where bonds are flexible. By providing open access to the database via ioChem-BD, we seek to accelerate progress in this field enabling systematic molecular dynamics studies for a broad range of POMs in solution accelerating the understanding of assembly, crystal growth, nanomaterials formation of metal-oxo clusters.

## Background & Summary

Polyoxometalates (POMs) are anionic polynuclear metal oxo clusters^1^ exhibiting a wide range of shapes and sizes^2–6^ due to nucleation and self-assembly processes^7^. Their high negative charge strongly influences their stability, which is dictated by interactions with their environment, including solvent^8,9^, counterions^10,11^, and other species in solution. Owing to their size and charge, POMs form non-covalently bonded complexes with a wide variety of chemical species, from cationic species (via strong ion-pairing with alkaline or organic cations) to neutral molecules such as small organics, proteins^12–15^, or even carbon nanotubes^16–19^. Additionally, POMs can be functionalized covalently or non-covalently with organic or inorganic moieties^20^, making them promising precursors for nano-structured materials^10,21,22^ with applications in fields such as photovoltaics^23–25^, catalysis^26^, medicine^14,27,28^, energy storage^29–31^, and material science^32^.

With the experimental community’s growing focus on POMs^33–40^, computational research has progressed in parallel. Over the past decade, the number of theoretical studies on POMs has steadily increased^41^. Density Functional Theory-based methods have facilitated unravelling their electronic structure^42^, acid-base properties^43^, ion-pairing^44^, as well as their spectroscopic^45^, electrochemical^46^, and reactive properties^47^. More recently, new methods enabled simulating POMs’ speciation thus offering predictive insights and understading on the nucleation mechanisms^48,49^. Dealing computationally with POMs is just complex. In addition to the intrinsic nature of highly negatively charged anions, particular counterions, solvent molecules and other species play a crucial role in real experiments. Explicitly accounting for the environment surrounding a POM in solution becomes a need since all those factors significantly influence their properties. As a result, computational research i nto the interactions between POMs and their environment in solution provides new opportunities.

For simulating this kind of systems at an atomistic detail, both solvent molecules and counterions need to be considered explicitly in order to capture dynamical phenomena such is ion-pairing. Classical molecular dynamics^50^ (MD) simulations are the commonly used method of choice. However, MD methods require the definition of forcefield (FF) parameters, which normally include bonding and non-bonding terms. Unfortunately, there is not a unique way to generate forcefield parameters for POMs. Accurately describing these systems with FFs is challenging, as metal-oxo clusters exhibit numerous oxidation states, binding and coordinations modes that are not observed in organic molecules, which makes it necessary to develop distinct FFs for each metal cluster.

The study of polyoxometalates has experienced sustained growth over recent decades^7,34,51–53^, driven by their broad structural diversity and wide range of applications in catalysis, energy storage, and biomedicine. In parallel, experimental characterization has expanded significantly, leading to an increasing number of structurally resolved and functionally diverse POM systems. Computational studies have grown alongside these experimental advances, particularly those based on electronic structure methods^33,41,53^, which have provided detailed insights into electronic structure, reactivity, and solution-phase behaviour. However, despite this progress, molecular dynamics simulations remain comparatively less developed and are typically restricted to specific systems and applications, such as ion pairing and interactions with biological environments^54–56^. This disparity highlights the need for reliable and transferable force-field parameters to enable systematic atomistic simulations of polyoxometalates in solution.

Following pioneering studies aimed at developing FF parameters for POMs^57–59^, MD simulations of POMs in explicit water including counterions were later introduced^60^, providing an important step towards a more realistic description of these systems in solution. That work served as reference to many other MD studies on POMs^61^. In those MD simulations, the polyanion was modeled as a rigid body with fixed intramolecular parameters, and electrostatic interactions were handled using charges derived from the molecular electrostatic potential (e.g., CHelpG) calculated via DFT. This methodology has yielded significant insights into POM chemistry. A series of studies by Chaumont and Wipff^62–65^ enabled highlighing the importance of the solvent (especially water) in POM POM interactions. Subsequent studies on lacunary Keggin anions^66^ and the aggregation patterns of Lindqvist salts^67^ demonstrated that higher negative charges lead to an increase degree of POM aggregation. Moreover, recent studies have shown the capability of MD simulations to describe the interactions of POMs with biological systems, such as proteins^54,68^. In the field of hybrid POMs, MD simulations have been used to describe the structural packing of hybrid organic POMs and the mechanisms underlying their supramolecular assembly^69,70^. Moreover, simulations of a hybrid POM decorated with light-harvesting antennas highlighted the importance of explicitly accounting for both solvent and cations in order to understand the corresponding electrochemical and electron-transfer processes^71^. Recently, the first MD study using flexible FF parameters for a POM was published by Sciortino *et al*.^72^. In this study, the authors rationalized the interactions of decavanadates and decaniobates with proteins in explicit water. The flexibility of the POM was achieved using MCPB.py^73^, a Python-based metal center parameter builder. This code, based on Seminario method^74^, computes force constants from the cartesian Hessian matrix obtained through a QM method. Later, we explored the Hofmeister effect in Keggin-type polyoxometalates^75^, employing flexible POM bond parameters derived from the modified Seminario method^76^. This approach allows the POMs to adapt to confined environments like cyclodextrin cavities. There has been a steady increase in the number of MD studies focused on POMs^56^, driven by the rising interest in developing new materials embedding POM moieties. These materials offer unique properties and promising applications across various fields of chemistry^77–80^. Consequently, there is a clear need for tools that simplify POM simulations set up, and also to provide standard and reproducible parameters. In response, we developed **POMsDB** aimed at facilitating advancements in molecular simulations in the rapidly growing field of metal-oxide clusters science.

To establish a standard for MD simulations of polyoxometalates, it is essential to accurately define the atom typing of the metal oxide clusters. Atom typing rapidly becomes extremely complex even for relatively simple molecules, serving as a lossy compression that reduces the detailed chemical environment into simplified labels, which can obscure critical chemical characteristics necessary for accurate parameterization. The process often involves subjective decisions, leading to inconsistencies and potential errors that propagate throughout the force field development process. Additionally, atom typing demands significant expertise, limiting the number of individuals or groups capable of developing or modifying force fields. To address these issues, we developed Polytype^81^ code which generates unique atomtype for polyoxometalates retaining detailed information about each atom.

MM-based methods for deriving force-field parameters for metal ions^82^ encompass cationic dummy-^83^, nonbonded-^84,85^, and covalent models^73,86,87^. The latter model explicitly considers bonds, angles, torsions, and van der Waals and Coulombic interactions between coordinating ligands and metal ions. Three common methods for deriving bond force constants include calculating the Hessian matrix, using energy-fitting method^88^ and force-matching^89^. Concerning Hessian-based methods (the one used in **POMsDB**), the Seminario method^74^ is the most widely applied. The Seminario method has been successfully utilized to model small metal-organic molecules within parameter derivation toolkits^90,91^, including development of Zn FF for metalloproteins^92^, metal-organic frameworks (MOFs)^93,94^, and metal-organic cages (MOCs). It parametrizes harmonic bond and angle force field parameters directly from the QM-derived Hessian matrix, thereby avoiding the need for empirical data. However, this method tipically excludes dihedral parameters involving metal ions, assuming they have minimal impact on the overall structure. Cole and colleagues^76^ further improved the method by addressing the issue of double-counting bending interactions, which resolves a key limitation of the conventional approach that tends to overestimate angle force constants, making them excessively rigid. This improvement is achieved by applying a multiplicative correction factor that accounts for the specific molecular environment. Despite its advantages, it is worth noting that this improved approach has not been widely adopted, as most MD tools embedded in software packages (such as the VFFDT plugin^87^ and the MCPB.py program^73^ in AmberTools) continue to rely on the conventional Seminario method. A recent promising automated tool using the modified Seminario method, named *Metallicious*^95^, has been introduced for parameterizing covalent metal models in supramolecular systems. However it only supports organometallic structures where metals are separated by at least two nonmetal atoms, and thus, metal clusters are not currently supported. More sophisticated force field potentials have been developed for metal ions, for instance Grimme *et al*. developed a generic force field that includes parameters for most metals, which has been effectively applied to gas-phase optimizations of MOFs and MOCs^96^.

Both the Seminario and Modified Seminario methods are largely independent of the underlying force field used to calculate torsional and non-bonded parameters. For our work, we have chosen the Modified Seminario method due to its improved performance compared to the original Seminario approach. In rigid molecules, such as polyoxometalates, bond and angle constraints will naturally enforce a geometry that indirectly restricts dihedral angles. Consequently, even when dihedral angles are not explicitly optimized by the Hessian matrix, the bond and angle terms help maintain the dihedrals in a physically reasonable configuration.

Given the structural diversity of polyoxometalates, it is important to contextualize the systems considered in this work. The selection of the 22 POM systems included in this first version of **POMsDB** was guided by the goal of covering a representative cross-section of the major structrual archetypes commonly encountered in POM chemistry^22,97,98^. The dataset spans multiple families (Keggin, Wells-Dawson, Lindqvist, and related metal-oxo clusters), while ensuring chemical diversity in terms of metal composition (including W, Mo, V, Nb), charge diversity across highly charged anionic species, and size diversity, ranging from small compact to larger clusters.

Importantly, these metals were specifically chosen because they correspond to the most commonly synthesized and experimentally characterized POM systems, which have been extensively studied due to their relevance in a wide range of applications, including catalysis, energy storage, and biomedicine. This ensures that the database is aligned with experimentally accessible systems and maximizes its applicability for both computational and experimental researchers^1,26,34,51,99^.

**Fig. 1:**
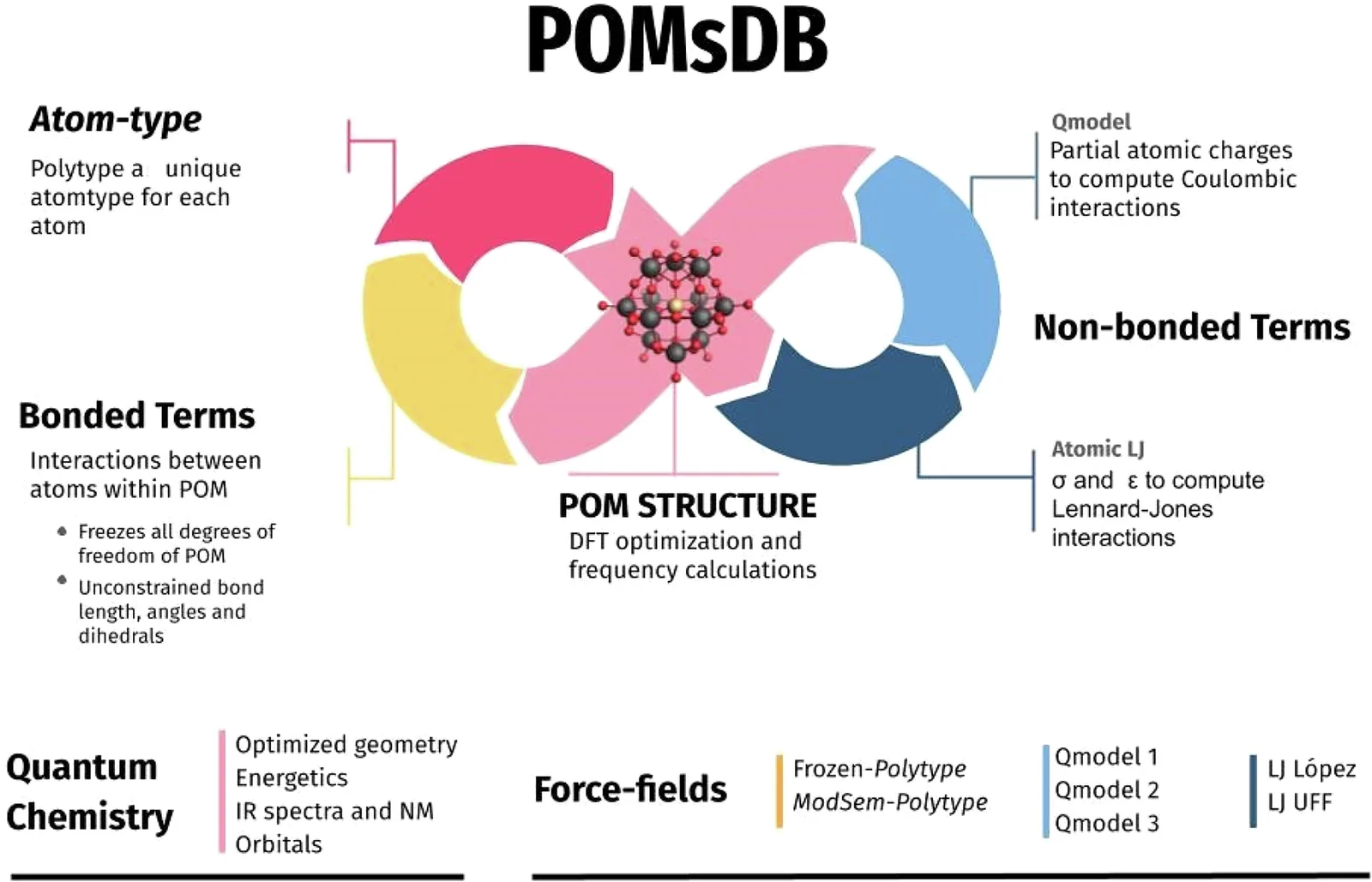
Schematic representation of the information contained in the **POMsDB** database. The force field is defined through a unified set of bonded and non-bonded interaction terms, parameterized according to atom type. At the bottom, the quantum mechanical data, together with the possible choices for the definition of bonded terms, partial atomic charges, and Lennard-Jones parameters.

**POMsDB** includes FF files for 22 POM molecules to run classical simulations. Frozen FF files are available for both AMBER^100^, and GROMACS^101^, whereas ModSeminario-derived FF files are currently distributed in GROMACS-specificic itp format only, although they are not directly AMBER-compatible and require conversion for use in AMBER. FF parameters in this database include atom typing, atomic point charges, Lennard-Jones (LJ) parameters, and equilibrium-bonded parameters (see Fig. 1). *Polytype* atom typing involves assigning a unique atom type to each atom, based on its atomic coordination number, while simultaneously classifying the oxygen atoms of the POM as terminal, bridging or central. Three distinct types of DFT-derived atomic charges and two sets of LJ parameters are available for selection. Additionally, two different definitions of intramolecular bonding parameters are provided: the standard approach used in previous MD studies of POMs, where the POM is treated as a rigid body (*Frozen-polytype*), and the approach based on the modified Seminario method^76^ (*ModSem-polytype*), where the POM behaves as a flexible object, allowing its intramolecular coordinates to evolve dynamically.

This paper presents **POMsDB**^102^, an open and freely accessible database of quantum-chemically molecular properties and force field parameters of 22 POMs. The name “**POMsDB**” was adopted to highlight that we aim to collect data on the chemistry of POMs. The database is available from the ioChem-BD platform^103^ which serves as a repository for sharing computational data together with several tools to plot results and analyze calculations. *Polytype*^81^ atom typing and generation of different bonded and non-bonded force field files were introduced. **POMsDB** includes all combinations. The possible selections are summarized in the bottom of Fig. 1. Two different bonded models, three charge models (Qmodels), and two LJ parameters sets could be selected leading to 12 classes of force field for each POM, so that the user can decide, according to the user’s criteria criteria, the combination of parameters that he/she considers most appropriate. This dataset facilitates performing MD studies of POMs in solution, and aims at accelerating the understanding of dynamic phenomena of metal-oxo clusters.

## Methods

### DFT calculations: geometric structure and vibrational analysis

Atomic coordinates were obtained by full geometry optimization with the Gaussian09^104^ software package or ADF2019^105^ using the density functional theory (DFT) GGA functional BP86^106^. In Gaussian09, the def2-TZVP^107^ basis set was used, while in ADF the standard Slater-type Triple-ζ plus polarization basis (TZP)^108^ was selected, along with relativistic effects accounted for using the scalar ZORA approach^109^. All calculations incorporated solvent effects, i.e, the Polarized Continuum Model^110^ applied in Gaussian, and the COSMO model^111^ in ADF. We ascertained that none of the optimized geometries contained imaginary vibrational frequencies. We developed a set of scripts, called *AutomatedPOMsDB*^112^, that collects molecular properties (optimized atomic coordinates, energies, IR frequencies, atomic charges) and force-field (FF) parameters, and generates all the files needed to run molecular dynamic (MD) simulations in common MD codes.

### Forcefield parameters

#### Atom typing

We have developed a new systematic method for atom typing in POMs, called *Polytype*^81^. This tool assigns a unique label to each atom based on its local connectivity, distinguishing between terminal, bridging, and central oxygen atoms, while also including the coordination number of each metal center. For example, in the system [AlMo_12_O_40_]^5−^, the label *OB2017* represents a bridging oxygen coordinated to two atoms, with position 17 in the atoms list, while *Mo6001* corresponds to an octahedrally coordinated molybdenum at position 1.

This approach enables automated force-field generation while preserving the chemical heterogeneity inherent to polyoxometalate structures. Unlike conventional atom-typing schemes, which often rely on simplified classification rules, Polytype assigns a unique atom type to each atom, allowing the force field to explicitly represent this intrinsic chemical heterogeneity. This atom-specific resolution is essential for capturing subtle variations in local coordination environments that are otherwise lost in conventional atom-typing approaches. As a result, the need for manual intervention is greatly reduced, while consistency across different POM structures is maintained. This is particularly important for scalable force-field development and for future extensions to mixed-metal systems.

#### Bonded parameters

No standardized method exists for generating bonded parameters for POMs. Starting from the optimized geometric structure, we then define two classes of bonded parameters, termed **Frozen-Polytype** and **ModSem-Polytype**.

- **Frozen-Polytype**. At the optimized geometry, a harmonic potential with a strong distance constraint is imposed, using a high spring constant of 10^6^ kJ mol^−1^ nm^−2^ between each atom pair within the molecule. This ensures that each atom in the molecule remains constrained within a specific distance range relative to every other atom in the molecule, creating a rigid, compact structure. During the simulation, the POM is able to move and rotate freely within the simulation box while preserving the original DFT-optimized geometry.
- **ModSem-Polytype**. Starting from the optimized DFT geometry, the cartessian Hessian matrix was used to calculate bond and angle force constants through the Modified Seminario method^76^, employing the Modified Seminario program available on GitHub (https://github.com/aa840/ModSeminario_Py).

### Non-bonded parameters

#### Partial atomic charges

The most crucial step in generating a forcefield for POMs is accurately describing the Coulombic interactions within the metal-oxo cluster. This is essential because POMs are highly charged anionic species, making electrostatic interactions the primary mode of interaction with the environment. **POMsDB** includes the three atomic charge models most commonly used in the literature. All charges were calculated from the optimized structure at the DFT level using the BP86 functional and the triple-ζ def2-TZVP basis set. Table 1 summarizes the three models provided.

**Table 1:** Schematic description of the DFT level and charge derivation scheme used in the charge models (QModels).

	QModel 1	QModel 2	QModel 3
**DFT level**	BP86/LANL2DZ	BP86/def2-TZVP	BP86/TZP
**Charge model**	RESP (CHelpG)	RESP (CHelpG)	Multipole (MDC-q)
**Atomic radii**	Metal: crystal Shannon radii; Oxygen: 1.52 Å	Metal: crystal Shannon radii; Oxygen: 1.52 Å	All atoms: Klamt

#### Atomic Lennard-Jones parameters

Atomic Lennard-Jones (LJ) parameters are assigned at the atomic level. For each pair of atoms *i* and *j*, the mixed LJ parameters *σ*_*ij*_ and *ε*_*ij*_ are then obtained using the Lorentz–Berthelot combination rules.

Two alternative LJ parametrization strategies are provided to reflect different approaches used in the literature and in the development of POM force fields:

UFF is a generic force field that enables a fully consistent and transferable description across all metal centers. This is particularly relevant when extending parametrization beyond the original López framework to metals not explicitly covered in earlier POM force fields.

- **LJ López**. This set follows the parameters employed by López *et al*.^60^, with extensions to additional elements according to the protocol used in subsequent studies. In this approach, Rappé UFF^113^ Lennard-Jones parameters are assigned to atoms other than P, W, and O^114,115^.
- **LJ UFF**. In this set, Rappé UFF Lennard-Jones parameters are assigned to all atom types, ensuring full internal consistency across the force field. Although UFF is a generic force field and does not explicitly account for the full complexity of metal–oxygen interactions in highly charged clusters, its use enables a consistent and transferable description across all metal centers. This is particularly relevant when extending parametrization beyond the original López framework to metals not explicitly covered in earlier POM force fields.

## Data Records

The dataset is available through the *ioChem-BD* web interface, which provides an intuitive and structured environment for accessing all data associated with **POMsDB**.

**Fig. 2:**
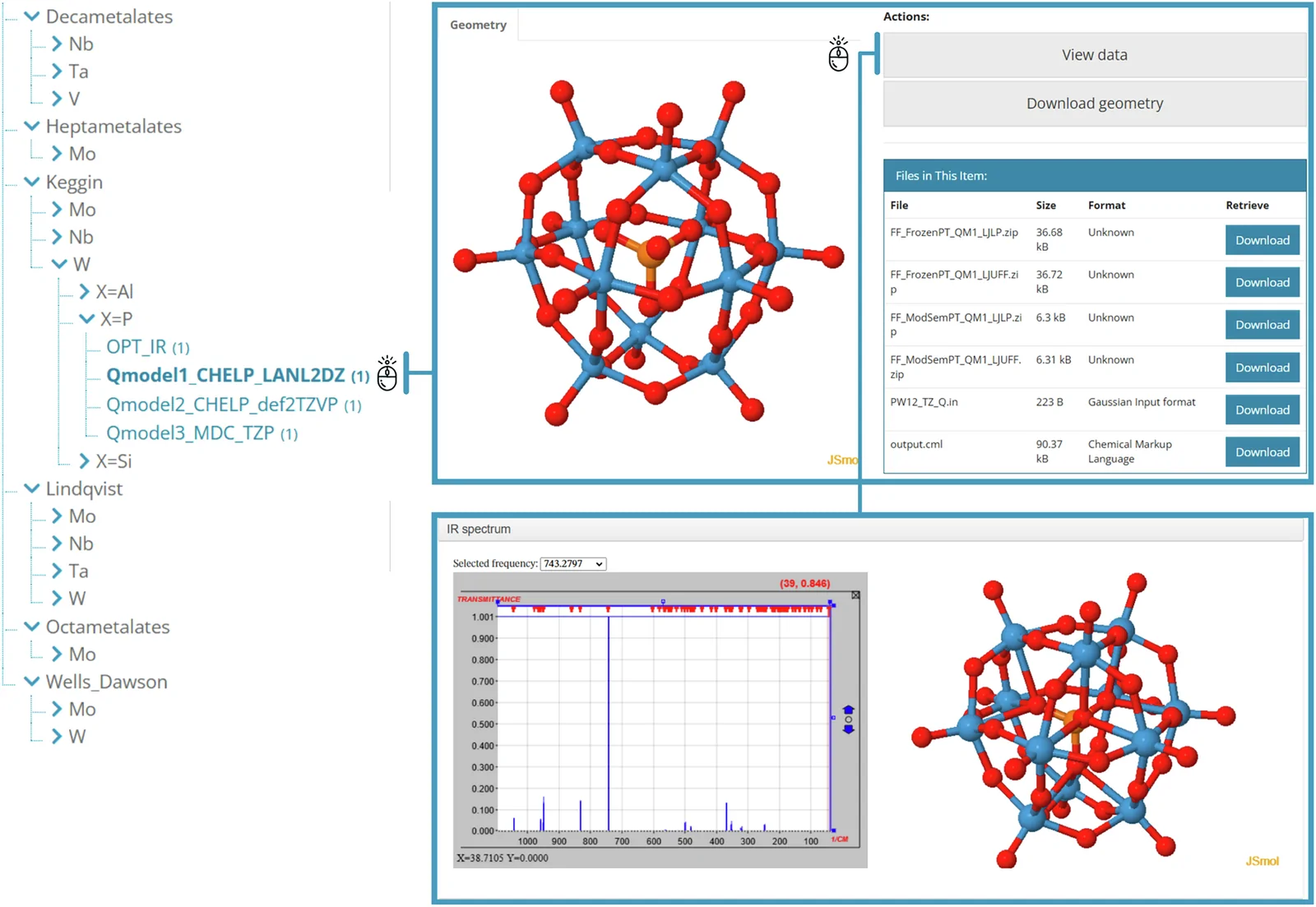
Graphical Interface of **POMsDB** in *ioChem-BD*, showing an example of [PW_12_O_40_]^3−^. The *ioChem-BD* platform provides access not only to the force field parameters for the entire series of calculated POMs but also to other relevant properties. These include molecular orbital energies and simulated IR spectra among others, offering a comprehensive resource for exploring the characteristics of polyoxometalates.

On the main page of **POMsDB** (see Fig. 2), a hierarchical tree of molecules is displayed on the left. For each polyoxometalate system, the database provides complete sets of input and output files from Gaussian and ADF calculations, including geometry optimizations and frequency analyses, as well as the three QModel charge calculations.

By accessing the “view data” interface, users can retrieve a wide range of molecular properties, including optimized atomic coordinates, electronic energies, enthalpies, Gibbs free energies, molecular orbital energies, Mulliken charges, ESP (CHelpG) charges, and vibrational spectra.

In addition, force-field parameter files are provided in formats compatible with common molecular dynamics packages. These include bonded interaction parameters and non-bonded terms. All force-field files for each system can be downloaded as a compressed archive (ZIP file) directly from the corresponding dataset entry.

### Usage notes and parameter selection

**POMsDB** is designed as a multi-level parametrization framework rather than a single force-field prescription. In the current state of the field, there is no universally accepted “best” force-field combination for polyoxometalates, in a similar way to how different DFT functionals are routinely selected depending on the system and target property. Instead, the available combinations reflect alternative, literature-established parametrization strategies that have been successfully employed in previous molecular dynamics studies of POMs.

For this reason, **POMsDB** provides twelve internally consistent force-field combinations, enabling users to select the parametrization that best matches their scientific objectives, level of accuracy, and desired continuity with prior literature. This design ensures reproducibility while avoiding the adoption of a single methodological choice that could bias future applications or limit methodological flexibility in this rapidly evolving field.

The available options combine three charge models, two Lennard-Jones parameter sets, and two bonded interaction schemes (rigid and flexible representations of the POM core). The charge models provide alternative quantum-chemical descriptions of the electrostatic potential, while the Lennard-Jones parameter sets reflect either continuity with previously reported POM force fields or full internal consistency using a single parameter source. The bonded models determine whether the POM is treated as a rigid entity or as a flexible structure derived from the modified Seminario approach.

Overall, the choice of parameters should be guided by the specific application and modelling goals, rather than a single optimal prescription. This framework is intended to reflect the diversity of established modelling practices in the literature.

## Technical Validation

### Validation of QM results

All 22 polyoxometalates were modeled using standard quantum chemical methods for geometry optimization and vibrational frequency calculations. The BP86 functional, commonly employed in the theoretical description of polyoxometalates due to its excellent performance in predicting metal-oxide geometries and properties^41^, together with the def2-TZVP basis set^107,116^, was used throughout this work. All optimized structures were confirmed to correspond to true minima on the potential energy surface, as verified by the absence of imaginary frequencies. Vibrational frequencies were computed at the same level of theory to ensure consistency between structural and spectroscopic properties.

In order to further validate the reliability of the quantum mechanical (QM) approach, both structural and vibrational benchmarks were performed against experimentally characterized reference polyoxometalates, namely the *α*-Keggin anion [PW_12_O_40_]^3−^ and the Lindqvist-type clusters [Nb_6_O_19_]^8−^ and [W_6_O_19_]^2−^. These systems provide complementary validation across complex heteropolyanions and highly symmetric metal-oxide clusters.

Computed interatomic distances are in excellent agreement with crystallographic and spectroscopic data reported in the literature (Table 2). For the *α*-Keggin anion, the calculated terminal W = O distance (1.72 Å) and the bridging W–O–W bonds (1.94 Å) deviate by less than 0.03 Å from the reference X-ray structures^117^. Similarly,the Lindqvist clusters reproduce the characteristic metal-oxygen framework with high fidelity, capturing the subtle differences between the Tungsten and Niobium coordination spheres. The overall Mean Absolute Deviation (MAD) for bond lengths across all benchmark systems is approximately 0.03 Å (computed using averaged experimental reference values), confirming that the BP86/def2-TZVP level of theory provides a robust and reliable foundation for the subsequent generation of force field parameters and the description of structure of polyoxometalates.

**Table 2:** Validation of DFT-optimized structural parameters for representative polyoxometalates. Experimental reference values are compared with results obtained at the BP86/def2-TZVP level of theory.

System	Parameter	Experimental	POMsDB (DFT)	Ref.
*α-[PW*_12_*O*_40_*]*^3−^*(Keggin)*	W = O_*t*_ (Å)	1.69^*a*^,1.70^*b*^	1.72	^*a*^^119^^,^^*b*^^120^
	W–O_*b*_ (Å)	1.94^*a*^,1.90^*b*^	1.94	^*a*^^119^^,^^*b*^^120^
	W–O_*c*_ (Å)	2.43^*a*^,2.44^*b*^	2.48	^*a*^^119^^,^^*b*^^120^
*[W*_6_*O*_19_*]*^2−^*(Lindqvist)*	W = O_*t*_ (Å)	1.69	1.73	^121,122^
	W–O_*b*_ (Å)	1.92	1.94	^121,122^
	W–*μ*_6_-O (Å)	2.33	2.36	^121,122^
*[Nb*_6_*O*_19_*]*^8−^*(Lindqvist)*	Nb = O_*t*_ (Å)	1.77	1.83	^121,122^
	Nb–O_*b*_ (Å)	2.01	2.02	^121,122^
	Nb–O_*c*_ (Å)	2.38	2.40	^121,122^

Beyond the structural validation, where computed bond distances show high consistency with X-ray data, a comparison of vibrational frequencies was performed to assess the dynamical description of the clusters (Table 3). It is important to note that frequency comparison is particularly delicate in polyoxometalates; while our calculations are performed in a simulated aqueous phase, experimental benchmarks are often obtained from solid-state spectra. In the crystal lattice, vibrational modes are significantly constrained by intermolecular interactions and the direct proximity of counterions, which are more influential than in a continuum solvation model.

**Table 3:** Validation of DFT-computed vibrational frequencies for representative polyoxometalates. Experimental reference ranges are compared with results obtained at the BP86/def2-TZVP level of theory.

System	Mode	Experimental (cm^−1^)	POMsDB (DFT)	Ref.
*α-[PW*_12_*O*_40_*]*^3−^*(Keggin)*	*v*(W = O_*t*_)	960–1000	959–976	^118^
	*v*_*as*_(W–O–W)	850–890	838–859	^118^
*[W*_6_*O*_19_*]*^2−^*(Lindqvist)*	*v*(W–O–W)	812-840	744–779	^123^
	*v*(W = O_*t*_)	975–1000	942–971	^123^
*[Nb*_6_*O*_19_*]*^8−^*(Lindqvist)*	*v*(Nb–O–Nb)	685	633–675	^124^
	*v*(Nb = O_*t*_)	856	746–755	^124^

Despite these environmental differences, the results successfully reproduce the fundamental spectroscopic trends between different metals and frameworks. For instance, the computed *v*(W = O_*t*_) for [PW_12_O_40_]^3−^ at 959–976 cm^−1^ falls precisely within the experimental range of 960–1000 cm^−1^^118^ without the need for empirical scaling factors. Although the bridging *v*(M–O–M) frequencies are systematically underestimated, as expected in the absence of crystal lattice constraints, the calculations correctly reproduce the redshift observed when moving from Group 6 (W) to Group 5 (Nb) metals. These results indicate that the BP86/def2-TZVP level of theory provides a physically meaningful description of bond stiffness and of the underlying electronic trends across the POMsDB database.

### Validation of forcefield parameters

Among the properties related to polyoxometalate dynamics in solution, self-diffusion coefficients provide one of the most direct quantitative links between molecular dynamics simulations and experiment. Table 4 therefore compares previously reported experimental and computational diffusion coefficients for [PW_12_O_40_]^3–^ in water with the values obtained using **POMsDB**. All reported values fall within the same order of magnitude, and the diffusion coefficients obtained with **POMsDB** are consistent with the experimental range.

**Table 4:** Comparison of previously reported diffusion coefficients for [PW_12_O_40_] and **POMsDB** QModel 2 results obtained using rigid intramolecular POM coordinates (Frozen-Polytype) and flexible intramolecular POM coordinates (ModSem-Polytype).

[PW_12_O_40_] (mM)	Medium	*D* (10^−6^ cm^2^ s^−1^)	Technique
*Experimental*			
°0.05–5	H_2_O + 1 M H_2_SO_4_	3.36	Polarographic method^125^
°94	H_2_O	4.6	PGSE-NMR^126^
°30	H_2_O + NaCl	3.25	POSY-NMR^127^
*Computational*			
°*not specified*	H_2_O + Na^+^	2.4	MD (QModel 1), Lopez *et al*.^60^
°54	H_2_O + Na^+^	2.0	MD (QModel 1), Leroy *et al*.^61^
°150	H_2_O + ${{\rm{H}}}_{5}{{\rm{O}}}_{2}^{+}$	5.59	MD, Chaumont *et al*.^62^
°158	H_2_O + Na^+^	5.7	MD, Chaumont *et al*.^65^
**POMsDB**			
°50	H_2_O + Na^+^	2.6	MD, Frozen-Polytype (QModel 2)
°50	H_2_O + Na^+^	5.9	MD, ModSem-Polytype (QModel 2)

Diffusion coefficients were computed every 100 ps using frames separated by 10 ps, and the reported values correspond to averages over the sampled frames.

In addition to the good agreement found for diffusion coefficients, force-field performance for POMs in solution can also be assessed through structural descriptors that are well established in previous MD studies, such as hydration-shell organization, radial distribution functions, coordination numbers, and ion-pair populations^60,61,67,75^. For Keggin anions, these studies showed a marked heterogeneity of the hydration shell, with terminal oxygen atoms being more exposed to water and bridging oxygen atoms forming longer-lived contacts^60,61^. They also showed that counterion effects cannot be reduced to simple Coulombic attraction, since both contact and solvent-shared ion pairs contribute to the observed dynamics^61,67^.

However, unlike self-diffusion coefficients, these structural observables are generally not available as directly comparable macroscopic experimental quantities across a broad range of POM systems and conditions. For this reason, in the present work we use diffusion as the primary quantitative benchmark for force-field validation, while the broader microscopic behaviour captured by MD is discussed in the context of previous literature.

## Conclusion

In this work, we present **POMsDB**, an open and freely accessible database that provides quantum-mechanical molecular properties and force-field parameters for a representative set of 22 polyoxometalates. The database includes optimized geometries, energies, vibrational spectra, atomic charges, and bonded and non-bonded force-field parameters, all generated through consistent and reproducible computational workflows. By combining different charge models, Lennard-Jones parameter sets, and bonded interaction definitions, **POMsDB** offers a flexible framework that allows users to select the parametrization best suited to their molecular dynamics simulations.

The systems included in **POMsDB** were carefully selected to cover the most relevant structural families of polyoxometalates, ensuring diversity in size, composition, and charge while focusing on experimentally accessible and widely studied compounds. This makes the database directly applicable to ongoing research in catalysis, materials science, energy storage, and biomedicine.

The growing interest in polyoxometalates has led to a significant increase in computational studies; however, molecular dynamics simulations of these systems remain comparatively limited because of the challenges associated with accurate force-field parametrization. In this context, **POMsDB** addresses a critical gap by providing standardized and ready-to-use parameters, thereby facilitating the simulation of POMs in solution, including the explicit treatment of solvent and counterions. This is essential for capturing key phenomena such as ion pairing, self-assembly, and interactions with complex environments.

Emerging machine-learning interatomic potentials, such as UMA, MACE- or NequIP-based approaches, represent a promising complementary direction for the simulation of specialized systems, including polyoxometalates. Their application to highly charged POM clusters, particularly in solution, however, requires dedicated training datasets and validation protocols tailored to this chemical space. In this context, the primary contribution of **POMsDB** is to provide transparent, QM-derived, and readily deployable classical force-field parameters and molecular data, while also offering a structured foundation that may support future data-driven developments for POM simulations.

Overall, **POMsDB** represents a step forward in improving reproducibility and accessibility in the computational study of metal-oxo clusters. We anticipate that this resource will accelerate research in the field and provide a foundation for future developments. Because polyoxometalate chemistry is a highly active and continuously evolving field, with new structures being regularly synthesized and characterized, **POMsDB** is conceived as a dynamic and extensible resource designed to evolve alongside ongoing developments. Future work will focus on incorporating newly reported archetypal systems and on further expanding the chemical and structural diversity of the database, thereby enhancing its applicability and long-term value for the community.

## Data Availability

The dataset is available through the ioChem-BD platform at 10.19061/iochem-bd-1-224.
